# “Like a pickle that’s been unpickled”: Emotional, identity and behavioural transformations throughout hepatitis C treatment

**DOI:** 10.1371/journal.pone.0272401

**Published:** 2022-12-12

**Authors:** Stelliana Goutzamanis, Joseph S. Doyle, Danielle Horyniak, Peter Higgs, Margaret Hellard

**Affiliations:** 1 Disease Elimination Program, Burnet Institute, Melbourne, Victoria, Australia; 2 School of Public Health and Preventive Medicine, Monash University, Melbourne, Victoria, Australia; 3 Department of Infectious Diseases, The Alfred and Monash University, Melbourne, Victoria, Australia; 4 Behaviour and Health Risks Program, Burnet Institute, Melbourne, Victoria, Australia; 5 Department of Public Health, La Trobe University, Melbourne, Victoria, Australia; Johns Hopkins University School of Medicine, UNITED STATES

## Abstract

**Background:**

Little is known about the emotional experience and benefits of undertaking direct acting antiviral (DAA) treatment for hepatitis C. A better understanding of individual treatment outcomes can inform acceptable treatment delivery and promotion. We aimed to explore participant-perceived emotional benefits and transformations throughout DAA treatment among people who inject drugs, who were initiating treatment.

**Methods:**

Participants were recruited from either a community based clinical trial or community health clinics. Semi structured interviews were conducted with each participant before, during and following treatment. Interviews focussed on treatment perceptions, physical and mental wellbeing and modifiable health behaviours. Interviews were recorded, transcribed verbatim and thematically analysed. Participant and cohort matrices were produced to assess at which time point themes were present and whether themes changed or remained stable over time.

**Results:**

This paper presents analysis from 19 participants interviewed between 2017–2019. Most participants were male, with no or mild fibrosis. At baseline, all but one participant had injected drugs in the past month. Three themes relating to the emotional wellbeing and behaviour change described a common treatment experience; ‘hopes for better wellbeing’, ‘lifting the weight’ and ‘closing the chapter’. Participants were hopeful treatment would improve their emotional wellbeing. Hopes were actualised during treatment as participants began to feel uncertainty and stress easing. Completing treatment improved some participants perceptions of self. Some participants consciously changed their injecting behaviours during treatment.

**Conclusion:**

Undertaking and completing treatment was an emotionally and behaviourally transformative period. Participant perceived benefits should be used to inform how treatment benefit is conceptualised and how treatment is promoted in primary care settings.

## Introduction

Hepatitis C is an infectious viral disease primarily affecting the liver and most often affecting the lives of people with a history of injecting drug use [1]. In addition to hepatic fibrosis, hepatitis C may incur a significant but variable physical and emotional burden for those living with hepatitis C [2–4]. Fortunately, the introduction of curative direct acting antiviral (DAA) treatment has made individual cure a reality and the elimination of hepatitis C as a public health threat a possibility [5]. Nevertheless, meeting elimination targets requires increased rates of testing and treatment initiation [6, 7]. Studies exploring people’s attitudes towards, perceptions and experiences of DAA treatments will be crucial in shaping acceptable and effective treatment promotion.

In contrast with previous interferon-based therapies, DAAs are relatively straightforward, of short duration, highly efficacious and well tolerated [8–11]. Historically, interferon-based treatments required up to 48 weeks of medication, with complex dosing, low efficacy and a litany of intense physical and psychological side-effects [8, 11, 12]. Side effects included muscle and joint pain, insomnia, fever, chills, changes in mood and concentration [13, 14]. The arduous nature of treatment contributed to low uptakes rates [15]. Narratives of undertaking interferon treatment are marked by “horror stories” [16, 17], coping strategies within and outside of the clinical sphere [9, 17] and a display of determination and resilience [14, 18–21]. Although the extensive body of literature on the interferon treatment experience is now of diminishing relevance, it offers insights into how the legacy of interferon treatment may impact people’s engagement with DAAs.

There is an emerging but limited body of qualitative literature on the lived experience of DAA treatment. One qualitative study reported that how hepatitis C is perceived by those living with it has been consistent across both the DAA and interferon eras, illustrated by a persistent sense of cautiousness and uncertainty around treatment decision making [22]. Other early studies suggest the spectre of interferon treatment endured into the beginning of the DAA era, influencing perceptions of and willingness to initiate DAA treatment [16, 23]. In Australia, heavily subsidised DAA treatment has been widely available to all hepatitis C positive people in community based settings since 2016 and we are now beyond the introductory phase of DAA treatment [24]. It was estimated that between 2016 and 2018 a third of people living with chronic hepatitis C in Australia initiated treatment, many of whom were people with a history of injecting drug use [6, 25]. It is unclear whether peoples’ beliefs, perceptions and knowledge of DAA treatment has evolved since the early roll-out of DAA treatment.

Contemporary hepatitis C treatment literature is predominated by the population level benefits of DAAs. Metrics around incidence, transmission and cost effectiveness are used to assess the impact of DAAs and inform cost-effectiveness models [24, 26–28]. These metrics contribute to a dominant discourse that conceptualises the benefit of treatment as clinical and epidemiological [29]. Similarly, public health messaging often promotes treatment as an opportunity for cure and improved liver health. Individual level outcomes have mostly been measured using generic health-related quality of life scales [26, 30, 31]. There has been little qualitative investigation to provide context to quality of life studies and illuminate the breadth of self-described benefits of treatment, particularly social and emotional benefits. This longitudinal qualitative study aims to explore emotional experiences of DAA treatment before, during and following treatment among people who inject drugs.

## Methods

### Study design

We used longitudinal qualitative research methods because perceptions of healthcare can change over time and retrospective accounts of treatment don’t always accurately capture the treatment trajectory [32]. Gathering biographical data at multiple time intervals from the same participants introduces a unique level of temporality and complexity to understanding the lived experience. This paper draws on data from a longitudinal qualitative study, following 20 people throughout and following treatment, seeking to explore experiences of DAA treatment [33].

### Eligibility and sampling strategy

In order to recruit participants from diverse backgrounds and familiarly with research, participants were recruited from two different sources. First, the Treatment and Prevention (TAP) Study, a nurse-led community-based DAA treatment trial, assessing the feasibility of treating people who inject drugs and their injecting networks (clinicaltrials.gov identifier; NCT023363517) [34, 35]. The second recruitment source was two large community health clinics. Participants were eligible for this qualitative study if they were 18 years or older, self-identified as currently, or having a history of injecting drug use and were living with hepatitis C at the time of recruitment. Participants were also required to be in the process of initiating DAA treatment (either have a script or have had a treatment consultation). Sampling was opportunistic, with all eligible participants approached by TAP Study nurses or a general practitioner from the community health sites and informed about the qualitative study.

### Data collection

Data collection occurred between September 2017 and July 2019. Semi-structured interviews were conducted by the first author (SG) with each participant before, during and following DAA treatment. Interviews were conducted across seven different metropolitan Melbourne field-sites, in a range of quiet locations such as parks, cafes, private meeting rooms or the TAP Study van. Field-sites were places where Burnet fieldworkers and TAP Study nurses had an established presence and worked closely with local health and harm reduction services. Interviews occurred during the day and were often organised around clinical appointments or TAP Study visits. Basic demographic details were collected prior to the interview for participants recruited from health clinics. For TAP Study participants, this data was extracted from their TAP Study screening survey. Interviews were flexible and conversational, focussing on how treatment was constructed and experienced at each time point. Each timepoint (pre-treatment, during-treatment and post-treatment) had separate but similar loosely structured interview guides (S1 File). The interview guides were all developed prior to data collection. In order to capture change over time, key topics were included at all timepoints, these included; treatment perceptions, treatment knowledge, physical and emotional wellbeing, social support, modifiable health behaviours, treatment logistics and attitudes towards reinfection. Some topics related to a single time point. For example, sources of information, diagnosis experience, treatment motivations, and the process of treatment initiation were only discussed in the pre-treatment interview. Interview transcripts were read prior to follow-up interviews so participants could be asked about specific experiences previously mentioned (for example previously stated intentions to reduce alcohol consumption or start exercising). Interviews lasted on average 40 minutes (range: 17 minutes—70 minutes) and were recorded with a handheld digital voice recorder.

Reflexive discussion took place between the first and senior authors following each interview. The first author kept a reflexive journal throughout the study to document beliefs, rationales for methodological decisions, assumptions and reflections on interviews, as well as how understanding of study topics changed over time.

It should be noted that the lead author does not share a lived experience of hepatitis C. Further, the research team share the opinion that DAA treatment is beneficial and should be made globally freely available. Although questions were open ended, broad and focussing on the holistic treatment experience, it is possible pre-existing beliefs influenced the interviewing process by placing greater emphasis on follow up questioning when participants mentioned positive treatment outcomes.

### Data processing and analysis

Two stages of analysis were performed. Firstly, a thematic analysis [36] was used to inductively identify themes relating to shared experiences and understandings of treatment. Secondly, a longitudinal trajectory analysis [32] was performed to explore whether themes changed over time. Analysis was conducted using NVivo (Version 12, QSR International, Australia).

Interviews were transcribed verbatim using Microsoft Word and Windows Media Player following each interview. Identifying information was removed, participants were given pseudonyms which are used throughout the paper and initial thoughts were recorded. Once all data had been collected, inductive open coding began. All interviews from one time point were coded before coding the subsequent time point. Codes were short words or phrases capturing a single facet of semantic or latent meaning (e.g. “fear of end stage liver disease”). Codes relating to similar broader concepts were collapsed to generate initial themes, that represent patterns of shared meaning (e.g. “lifting the weight”). Themes were then reviewed, revised, named and defined and a thematic map produced. This process of analysis was reflexive, representing an interpretation of participant data. As such we did not seek to create a sense of objectivity through cross-coding and coding frameworks. However, themes were discussed among co-authors and colleagues who work with people who inject drugs around the field site areas.

Longitudinal analysis involved using Saldana’s 16 questions for longitudinal data (e.g. “what contextual and intervening conditions appear to influence and affect participant changes through time?”) [37]. Tables were produced for each participant and the cohort to describe at which time point themes are present, whether themes remain stable or transform over time, what contextual factors influence themes and whether themes are related at different time points. Quotes and language from every participant are utilised in this paper and disconfirming cases are highlighted where possible.

### Ethics

Ethics approval for this study was received by the Alfred Hospital (243/17) and Monash University Human Research Ethics Committees. Participants provided written consent to all three interviews prior to the first interview, and further verbal consent in arranging subsequent interviews. Participants were reimbursed AUD$40 following each interview. Participant contact information was securely stored separately to interview and demographic data.

## Results

### Participants

Fifty-four interviews were conducted with twenty participants. Eleven participants were recruited from the TAP Study and nine from community health clinics. One participant was excluded from analysis for this paper as they spontaneously cleared their hepatitis C after the first interview and did not initiate treatment (but did complete subsequent interviews). Most (n = 15) participants completed all three interviews, four participants completed the first two interviews and only one participant completed one interview. Reasons for loss to follow up were disconnected phone, no mobile phone or moving rurally and unable to travel to Melbourne for interview.

Participant demographic characteristics are summarised in Table 1. Notably, most participants were male and had a low level of fibrosis (METAVIR score F0-F1). Participant hepatitis C histories were diverse. Most participants were first diagnosed upwards of ten years ago, but for one participant it was just a matter of weeks before the first interview. Most participants (n = 17) had never previously been treated with DAAs or interferon therapy. Two participants had previously completed DAA treatment but become re-infected and one participant had completed both interferon and DAA treatment but both prior treatment episodes did not cure their infection.

**Table 1:** Participant demographic characteristics at baseline (N = 20).

**Total participants**, n (%)	20 (100)
**Total interviews**, n	54 (90% response rate)
**Gender**, male, n (%)	14 (70)
**Age**, years, mean (range)	39.15 (20–54)
**Employment status**, unemployed, n (%)	18 (90)
**Country of birth**, Australia, n (%)	14 (70)
**Injected drugs in the past month**, n (%)	19 (95)
**Fibrosis status**	
None to mild	11 (55)
Moderate to severe	4 (20)
Cirrhosis	0 (0)
Don’t know	5 (25)

This paper focuses on emotional benefits and transformations from undertaking DAA treatment. The three themes presented chronicle common elements of the treatment experience for participants in our study. This begins with participants’ hopes for improved wellbeing, which are actualised as the emotional burden of living with hepatitis C beings to ease during treatment. Finishing treatment marks a transformation in identity and a more positive perception of self. This paper focusses specifically on emotional elements of treatment, however, it should be noted participants also reported a range of physical and cognitive benefits from treatment. These findings are reported elsewhere [38]. Further, many participants often could not categorise perceived benefits as exclusively physical or emotional as they were often intertwined and conceptualised by how they impacted everyday life and relationships with others.

### Hopes for better wellbeing

Participants’ emotional journeys throughout and following treatment began with cautious optimism that treatment would afford them modest and wide-ranging improvements in their physical and mental health beyond simply curing their infection. This sense of hopefulness and anticipation was strongly situated in the first, pre-treatment interview. How much and what participants hoped would change by undertaking treatment was influenced by prior treatment experiences, familiarity with interferon treatment, motivations for initiating treatment, how much they felt hepatitis C impacted their life and whether they were a parent. Desirable treatment outcomes fit into two categories: hopes for what the medication would deliver and hopes for what being cured of hepatitis C would deliver.

Most participants were hopeful the biomedical action of treatment would improve their physical health, in turn improving their everyday life and emotional wellbeing. This was prominent among participants (both male and female) caring for children and who had been living with suspected hepatitis C related fatigue for many years. This hope for improved energy levels was underpinned by often feeling too fatigued to take their young children to the park, on outings or play with them as frequently as they desired. Increased energy was perceived as a way to feel more in control of everyday life and ultimately to meet their own expectations of ‘good parenting’.

That’s what I’m hoping will happen; that I’m not so feeling sick all the time, a bit more energy and just feeling a bit better…I’ll be able to do more things with the kids, you know, not lie down so much in the afternoon and do more activities. (Miriam, female, 49 years old, pre-treatment)

Although almost all participants expressed hope for at least one personal benefit from treatment beyond achieving cure, some just wanted treatment to be a simple process with minimal side effects. One participant acknowledged that while they were excited about the prospect of treatment, they had more pressing social and health priorities and so comparatively treatment did not seem significant. For others, their hopes were influenced by the perception that hepatitis C did not have a large emotional or physical impact on their lives and that initiating treatment was opportunistic or convenient.

I just hope it just goes smooth you know, like three months or how long it takes, let’s hope it’s cleared up. One course of medication or (pause) I hope it doesn’t, I don’t have any side effects. That’s about all really. (Theo, male, 49 years old, pre-treatment)

How participants expressed their hopes for cure and how confident they were that these hopes would be realised seemed to be shaped, in part, by prior treatment experiences. Most participants had not previously undertaken any hepatitis C treatment but had lived with hepatitis C throughout the interferon era of treatment. For these participants an acceptable treatment had been long awaited, with many participants describing witnessing peers now experiencing severe liver complications of hepatitis C. As such, it seemed many participants had large but cautious hopes that treatment would be an opportunity for change and transition. Participants hoped to feel less stressed and worried and to regain a sense of ‘normality’ and motivation. Participants frequently expressed the desire that completing treatment would be a catalyst for improvement in other aspects of life with phrases such as; “hopefully things will come together after treatment”, “hopefully this will turn my life around a little bit” or “hopefully it just keeps getting better and better”. These hopes were, however, expressed with caution, uncertainty, and reservation. Interwoven with a general hopefulness was a narrative of self-reassurance or mental preparedness for if treatment was unsuccessful in providing cure and additional benefits.

I just hear that sometimes it doesn’t work for a certain person and I feel like that’s gonna be me, because that’s just my luck. But I know too that that’s just me. Look I’m hoping it does but if it doesn’t you can do it again; I believe you can do it again…I’m a bit like clumsy and full of bad luck and if it’s not going to work on someone it will be me haha, but that’s just me. (Simon, male, 40 years old, pre-treatment)

This led to heightened sense of self responsibility to avoid potential disappointment. Participants commenced treatment with a committed and proactive attitude, which was sustained throughout treatment.

I’m doing everything right, so (pause) yeah. I don’t want to be disappointed on my behalf, because of my actions. So if I’ve done everything right, then if it doesn’t work I know it’s not because of me, yeah. (Zara, female, 39 years old, during treatment)

It was often difficult to ascertain whether participants truly believed treatment may not be successful for them, or whether they believed they shouldn’t appear so outwardly confident in treatment, but rather, express tempered expectations to minimise potential disappointment. Participants were well versed in the distinction between interferon and DAA treatments, yet for older participants who had witnessed peers undergo interferon treatment, their hopeful attitude towards treatment outcomes was balanced with superstition. It seemed they were ‘hedging their bets’.

I really want it to work, but maybe if you want it too much you won’t get it, I don’t know. (Van, male, 44 years old, pre-treatment)

Whilst most participants were cautiously hopeful, one participant described having no expectations whatsoever for treatment. Kai has previously completed both interferon and DAA treatment, but neither were successful in curing his hepatitis C and so he was not confident he would experience cure during this treatment encounter.

But if I was to get rid of it, I would still hold that mentality that it might come back, so I’m not gonna be too hopeful about it, but not be so negative about. I’m just being like neutral. No expectations. (Kai, male, 37 years old, pre-treatment)

Conversely, Wesker, the youngest participant in this study, appeared the most optimistic and confident about treatment. Unlike most participants, he was diagnosed only a couple of months prior to the first interview and was offered DAA treatment upon diagnosis. Whilst he was highly informed on DAA treatment, he admitted to having little knowledge of interferon treatment and didn’t know anyone who had undergone interferon treatment, perhaps making him less cautious.

I mean because I’m expecting to be cured it won’t be like a surprise like; ‘oh my god I can’t believe it worked’ kind of thing. (Wesker, male, 20 years old, during treatment)

### Lifting the weight

Participants’ hopes that treatment would afford emotional benefits were largely actualised in subsequent interviews. All participants, irrespective of how long they had been living with hepatitis C, described some degree of improvement in wellbeing throughout or following treatment including improved mood, motivation, and confidence, as well as decreased internalised, anticipated and experienced stigma, uncertainty and stress. Most improvements in emotional wellbeing were attributed to participants simply knowing they had done something to cure their hepatitis C. Most notably participants described gaining an often-profound sense of release and relief during treatment, which culminated in a sense of achievement and motivation following treatment. In contrast to most participants, Alex nonchalantly described emotional benefits that were broad and either difficult to attribute to DAA treatment or perceived as minor in the scheme of his life.

Same old. Nothing too different but yeah, good. Emotionally it’s good, it feels good that it’s gone now. Normal health again, back to 100%. (Alex, male, 37 years old, post-treatment)

The most frequently reported benefit from undertaking treatment was the alleviation of stress and uncertainty associated with living with hepatitis C. This was characterised by the widespread description of undertaking treatment as a “weight off my shoulders”. The metaphorical ‘weight’ that participants consistently referred to seemed to be the element of living with hepatitis C that impacted them most. In this cohort the ‘weight’ of hepatitis C mostly related to the stress and uncertainty associated with social stigma, status disclosure, onward transmission and the long-term health impacts of living with hepatitis C. Regardless of whether the emotional burden of living with hepatitis C was in the forefront of participants minds or not prior to treatment, almost all participants described a surge of relief during treatment.

I really don’t want to be in pain in hospital and being hooked up to machines. Those sorts of things worry me, I’d rather not go through that. When you think about it, it is (pause) but you’ve got to just kind of put it out of your mind because then otherwise it will affect every day of your life. (Li, female, 46 years old, pre-treatment)

That’s a huge weight off your shoulders. Massive. So I think I’m definitely happier, partly because of that…not ending up in a hospital at the age of 70 on a machine. That’s huge. That was big worry for me always. Well, when I thought about it. (Li, female, 46 years old, during treatment)

In the pre-treatment interview many participants harboured internalised stigma and recounted recent experiences of discrimination, fear of judgment and fear of onward transmission. As such, participants had varying levels of comfort with disclosing their hepatitis C status. Several participants became increasingly more comfortable in disclosing their status once they had begun treatment. Participants took solace in being able to tell others they were on treatment whilst disclosing their status, because they expected to be met with less judgment and be perceived as proactive or ‘almost cured’. Finishing treatment brought a transformation in that participants no longer had hepatitis C to disclose or potentially transmit to others. This experience marked a newfound freedom and relief. Participants felt their interactions with others (injecting partners, healthcare workers, family, broader society etc) were no longer constrained by a persistent pressure of what they must, should or should not disclose about hepatitis C in different scenarios.

It’s a lot better for your life and people treat you different. Now I can say I’m hep C free. I don’t get that judgment of when I go ‘Oh yeah I’ve got hep C’ and they look at me like ‘ohh you’re a disease ridden person’, but it’s not like that. (Hans, male, 38 years old, during treatment)

It was somewhat liberating for participants to tell others they don’t have hepatitis C if they were asked.

Well the treatment is helping in a big way, because people always say like; ‘you’ve got something’ like when you’re a ‘junkie’ you’re always looked down on or like ‘you’ve got a disease’ but I haven’t anymore! That is great for me!…I can say; ‘fuck you, I’ve got nothing’ and I haven’t, and I won’t! So yeah. If someone says something now, I just say; ‘I haven’t shared’. (Van, male, 44 years old, during treatment)

### Closing the chapter and looking forward

Participants described feeling the emotional burden of living with hepatitis C begin to ease during treatment. For some this burden had fully lifted after completing treatment for some and for others it was after receiving confirmation of cure. Completing treatment and the lifting of this emotional weight brought a sense of renewal and achievement. Participants, particularly those who had sought treatment in the community or walked a long road to initiate DAA treatment were proud their actions had improved their physical or emotional health. This manifested in a more positive self-perception and the alleviation of some internalised stigma relating to hepatitis C.

It was good to know that I’d actually finished something. It was good to know that I’d actually achieved something for once that was actually worth achieving. (Simon, male, 40 years old, post-treatment)

Because I’m attempting to do something for myself I feel more positive, like when the hep C is gone, that’s another cancer I don’t have to worry about, because there is cancer on both sides of the family and I was always worried about liver cancer and stuff like that. (Cam, male, 54 years old, post-treatment)

Participants felt more confident and positive about their future. They felt they had gained momentum and started planning for their next steps. For example, several participants now felt confident to initiate or plan for future intimate relationships, something they were forgoing whilst living with hepatitis C.

Look, when I know for sure I’ve gotten rid of the hep C, it will open up a lot of things for me; because of having hep C I haven’t wanted to meet new women or sleep with people. So there is that stigma that I’ve always put on myself for having it. Whereas at the moment, I know I’m getting mentally better and treatment will work eventually, that I’m starting to plan for those sorts of things. (Finn, male, 41 years old, post-treatment)

The general attitude in the final interview was of having closed a small but significant chapter in life. This was embodied by a universal and strong desire to not become reinfected or to have to undergo treatment again. Closing this chapter was particularly important to several participants who were in the process of or wanting to stop their injecting drug use as they felt their experience living with hepatitis C was attached to their identity of someone who injected drugs.

It was good, it’s over. Hopefully I don’t have to go back and have anymore treatment and yeah it’s gone and I can just not think about it all the time and just move on. (Miriam, female, 49 years old, post-treatment)

Participants continuing to inject described having to navigate being at risk of contracting hepatitis C, for many, for the first time in decades. Whilst almost all participants had long been confident in their knowledge and practices to prevent infection, the idea of re-infection was for some, a new worry and concern. Since initiating treatment, some participants described developing a “healthy paranoia” or having a hyper vigilant attitude to avoid reinfection.

Believe me, I have been fucking super, super safe, you know what I mean. When I’ve used, you know what I mean. You know, like always using a new fit, everything, always new. You just don’t take any more chances; you know what I mean. It’s just so not worth it. So not worth it. (Simon, male, 40 years old, post-treatment)

Importantly, whilst participants had undergone the emotional transformations they hoped for, a few participants made the distinction that being cured of hepatitis C is not the same as never having had hepatitis C. One participant predicted they may encounter circumstances in healthcare settings where they are asked to disclose ever having had hepatitis C. Another participant noted they would not forget the emotional impact of living with hepatitis C.

I can put it behind me, but I won’t forget what has happened this whole time, like I started using needles and getting hep C and all that shit and going through the dramas, I’ll never forget that, nup. (Hugo, male, 34 years old, during treatment)

One participant summarised his transition from living with hepatitis C to being cured as “like a pickle that’s been unpickled” but went on to stress that this is not the same as a cucumber that has never been pickled.

For most participants ‘closing the chapter’ was an attitude of confidence, renewal and envisioned change that came with finishing treatment. However, a small subset of participants had adopted this attitude prior to initiating treatment and made multiple and large lifestyle and behavioural changes during the study. Changes included no longer using drugs, lowering their methadone dose, finding stable employment, consistent exercise and enrolling in educational courses.

One participant described having reached a critical stage of motivation prior to initiating treatment. Treatment was part of a larger set of objectives to achieve a desired level of stability for herself and her child. By the second interview she had enrolled in a course which she had begun by the final interview.

I feel like I’m at a turning point. No one’s making me do it; I’m just doing it for me and my son. Yeah, it’s taken a while, but I’m doing everything. (Zara, female, 39 years old, pre-treatment)

I’m progressing because I’m doing what I wanted to do. Before I didn’t have a course, I didn’t know which way direction, but I knew I wanted to do something that was possible and sustainable and lead me to a job. So this opens a door, opens a lot of doors really. (Zara, female, 39 years old, during treatment)

Two participants credited treatment for partially catalysing or consolidating decisions to change behaviours. Participants described gaining additional positivity and resilience from completing treatment that helped them to continue making changes in their lives. Prior to treatment Wesker had recently completed a detox program and was occasionally injecting drugs. He saw treatment as an opportunity to completely stop injecting drugs and the confirmatory cure blood test as an opportunity to reduce his alcohol consumption.

I want to completely stop using, I think, like I’m getting paid on Thursday and I have no plans to use, if I do it will be a point or two. I’ve been using a little bit here and there, but I think when I’m on treatment I don’t think I ever want to inject again. (Wesker, male, 20 years old, pre-treatment)

I haven’t been using, I haven’t used at all since the last interview and stuff. So you know I’m going good now. I’m not using. (Wesker, male, 20 years old, during treatment)

So yeah I have been coming down a bit now that I’ve booked this thing, this result [sustained virologic response blood test] I think I am gonna cut down more… it’s just one of those things where I’m like; ‘maybe I shouldn’t drink as much’ and I’m using nicotine patches as well, because there’s nothing that’s made me quit smoking, it’s just a thing like; ‘maybe I should’. I think it is kinda also the hep C medication thing and because I quit drugs. I kinda feel like if I’m doing a couple of good things right maybe I should do everything. I haven’t used since last time, but I don’t think I’ll use again. (Wesker, male, 20 years old, post treatment)

## Discussion

This study describes emotional experiences of DAA treatment for hepatitis C among a cohort of people who inject drugs. We present three themes (‘hopes for better wellbeing’, ‘lifting the weight’ and ‘closing the chapter and looking forward’), which chronicle a common treatment experience within our study. Participants described experiences of cautious hopefulness, followed by an easing of the emotional burden of living with hepatitis C, contributing to sense of renewal and accomplishment upon treatment completion. Participant narratives show that people who inject drugs who are undergoing DAA treatment want and do achieve wide ranging and profound emotional benefits from treatment.

Since their inception, DAA therapies have been heralded and promoted as a ‘cure’ for hepatitis C. Whilst treatments are curative for the vast majority of people living with hepatitis C, the concept of cure has been entrenched in a biomedical discourse [29, 39]. More broadly, the motif of cure has been critiqued for being predicated on returning an individual to their pre-illness state of health, which obscures the personal, social and emotional aspects of illness [40]. Rather, Warren and Addison [40] present a multidimensional conception of cure, in which being cured “shifts temporalities of life and of illness, prompts new engagements with risk, generates new socialities and materialities, reveals questions of equity and access, and transforms personhood”. Our findings support this more nuanced and holistic idea of cure and treatment benefit. Whilst a course of hepatitis C treatment is now only two to three months, treatment was a transitional period for participants in our study, marked by reconstituting identity in relation to hepatitis C. Participants described several changes when shifting from living with hepatitis C to being cured of hepatitis C. Firstly, participants had shifted from being able to transmit hepatitis C, to being able to become (re-)infected. This represented a shift in participants perceptions of being a potential ‘risk’ to others to being susceptible to infection. Having experienced living with hepatitis C the collective narrative was one of strong resolve to avoid reinfection and confidence in their knowledge to do so. Secondly, although completing treatment had allowed participants to regain the sense of ‘normality’ that they hoped for, they were met with a ‘new normal’, which was not the same as never having lived with hepatitis C. Despite cure bringing a sense of finality or closing of a chapter, some participants noted a history of living with hepatitis C would still be present in their memories or medical records. Finally, throughout treatment many participants developed a more positive perception of self, tied to decreased feelings of internalised stigma as well as feelings of accomplishment and pride in completing treatment.

Our findings highlight treatment as a transformative period, emotionally, behaviourally and for self-perception. Several prominent illness trajectory theories and conceptual frameworks have been used in the context of hepatitis C. Including concepts of ‘biographical disruption’ [41, 42], the ‘shifting perspectives of chronic illness’ and ‘chronic illness trajectories’ [43]. These frameworks and others describing patient trajectories are focussed on ‘critical junctures’ in the chronic illness journey and are most applicable to the experience of living with and managing illness [44]. There has been little theoretical focus on the transition from living with a chronic illness (hepatitis C) to no longer having that illness (achieving cure). Many participants in our study were diagnosed before any treatment options were available or when the treatment available was sub-optimal. Prior to initiating treatment some participants were under the impression they would die from hepatitis C and were navigating a traditional ‘chronic illness trajectory’. However, the advent of DAAs in Australia enabled participants to transition from chronic illness management to cure. Our research highlights a need to develop conceptual frameworks where cure is not an endpoint, but a physical, emotional and social transformation and an experience influenced by contextual factors such prior experience with interferon treatment, diagnosis in the interferon era and perceptions of hepatitis C.

Our findings are consistent with perceived emotional and psychological treatment related benefits reported in both the interferon [45] and DAA era [46–49]. However, the unique strength of these findings is in portraying a trajectory throughout treatment and capturing change within participants. Nonetheless, our study was not without limitations. Interviews were often scheduled before, between or following hepatitis C treatment related medical appointments. This was useful in situating interview discussion in the context of hepatitis C and treatment, but often limited the duration of interviews, meaning some concepts may not have been fully explored during interviews. Secondly, participants received a range of DAA treatments, which differed in duration and number of pills taken daily. This may have altered participant experiences with treatment. For example, those with longer courses of treatment may have found treatment more challenging or conversely more rewarding with a greater time for reflection on or observation of treatment benefits. However, this was not explored or compared in this study. Future research may wish to explore how treatment logistics (such as duration) and context influence the treatment experience. Finally, interviews and analysis were not guided by an existing theory of illness, wellbeing, or cure. This limits our findings from being used to critically examine a prevailing understanding of ‘cure’. Rather, findings should be interpreted as experiential and exploratory and inherently influenced by the authors’ assumptions of hepatitis C cure as beneficial.

The vast majority of participants described a life that was qualitatively different following treatment compared to before starting treatment. Cure is not simply a discrete biomedical endpoint. For participants of this study there was psychological and emotional value not just in being cured, but in completing treatment and even initiating treatment. These findings reflect the need for a greater emphasis on a patient-centred approach to treatment. Clinicians involved in hepatitis C treatment should go further than the biological conception of cure and promote the potential for variable personalised emotional and social benefits of treatment. This may provide new avenues for engaging people with treatment. A patient-centred approach to treatment would also mean that hepatitis C cure is not necessarily the end of care. Our findings demonstrate that people may feel motivated when beginning treatment or gain motivation throughout treatment. This presents an opportunity for treatment prescribers to offer holistic care and link people to services that may additionally assist them in achieving other health or life goals. Participants in our study readily expressed hopes for and experiences with treatment that reflected the social and emotional dimensions of living with hepatitis C. This conceptualisation of treatment should be echoed by how we perceive, promote and provide treatment.

## Supporting information

S1 File Interview guide. Interview guides are intended to be semi-structured and flexible. Topics, questions, and the order of topics varied across interviews.
(DOCX)
