# Supplementary material for: “Like a pickle that’s been unpickled”: Emotional, identity and behavioural transformations throughout hepatitis C treatment
Source: PLoS One. 2022 Dec 12;17(12):e0272401. doi: 10.1371/journal.pone.0272401 (PMC9744280; doi:10.1371/journal.pone.0272401)
Supplement: S1 File — Interview guides are intended to be semi-structured and flexible. Topics, questions, and the order of topics varied across interviews. (DOCX) [file pone.0272401.s001.docx]

**Semi-structured flexible interview guide for the pre-treatment interview**

| **Topic** | **Questions and prompts** |
| --- | --- |
| Introduction | So, to start with, how has your week been going?  *Prompts:* Do you want to just talk a bit about how things are going for you at the moment?  Has…impacted getting hep C treatment? |
| Attitudes towards and perceptions of treatment | How do you feel about starting treatment?  *Prompts:* In what ways do you think treatment will affect you?  Will treatment change anything for you? Emotionally? Physically? Socially?  Do you reckon you’ll feel different when you start?  Is there anything you are worried or excited about with treatment?  *Prompts:* What are you most looking forward to? Do you have any expectations of what treatment will be like? |
| Motivations | What made you want to start treatment?  *Prompts:* What does it mean for you to start treatment? Is it important to you? Why/why not? Where does hep C and treatment sit in your personal list of big life priorities? |
| Diagnosis experience and disclosure | Do you remember when you first found out you had hep C?  *Prompts:* Who told you? Where were you? Do you reckon you knew you had it before? Do you reckon you know how you got it? Is there a moment you can pinpoint that may have been how you got it? How long ago did you find out?  What did it feel like when you found out?  *Prompts:* What was your reaction when you found out?  Did you tell anyone you had it?  *Prompts:* Who do you tell? How do you feel about telling other people? In what circumstances would you tell people?  Does it come up in your circle of friends? Do people talk about it a lot?  Have you ever felt judged or like people treated you differently after telling someone?  Is it hard or easy for you to tell people about it? Why |
| Process of initiating treatment | So can you tell me a little bit about the process from when you found out to getting to this stage, where you are about to start treatment?  *Prompts:* Was it a long process? What did you have to do? When did you decide to get treatment? Where did you go? Did you have to get blood tests? How did you find getting the blood tests done? |
| Relationship with healthcare | Where do you usually go for your healthcare needs or if you need a script?  How do you think your relationship with healthcare is?  *Prompts:* Do you have a regular GP? Do you get along with them? Do you feel comfortable talking to them about hep C?  Have you ever had any negative experiences with healthcare? |
| Behaviours | Do you think knowing that you are about to start treatment you have changed anything in your life or any behaviours?  *Prompts:* Has anything changed recently with your diet? Exercise? Lifestyle? Smoking? Alcohol? employment or how you feel about working? How you interact with people socially?  Can you tell me a little more about…?  What made you want to change… now?  Was it difficult to change…?  Do you reckon you will do anything differently being on treatment?  Has the doctor told you anything you are meant to do while you are on treatment?  *Prompts:* Do you know what treatment entails and what you have to do? Do you reckon treatment will be tricky or easy? In what ways? |
| Information about treatment | What kinds of stuff have you heard about treatment?  Where did you first find out about treatment?  Where do you get most of your information about treatment? |
| Information sharing | Did anyone encourage you to start treatment?  Have you told anyone else about treatment?  How much do your peers and your circle talk about hep C?  *Prompts:* What do they say about it? What do you think about the stuff they’ve said? Why? If someone asked you about treatment, what would you say? |
| Impact of hep C | Do you notice hep C at all? In what ways?  *Prompts:* Do you reckon it impacts you physically? Emotionally? Socially? Can you tell me a little bit more about that?  Have you had the liver scan?  *Prompts:* How was that? How do you feel about your liver? Does it affect you? |
| Reinfection | Are you worried about reinfection at all? Why/why not? |
| Conclusion | Next time we chat you’ll be halfway through treatment, any thoughts on what you are hoping that will be like?  Anything else you want to chat about? |

**Semi-structured flexible interview guide for the during-treatment interview**

| **Topic** | **Questions and prompts** |
| --- | --- |
| Introduction | So, to start with, how has your week been going?  *Prompts:* Do you want to just talk a bit about how thing are going for you at the moment?  Has anything changed for you since last time we spoke?  *Prompts:* Can you tell me a bit more about…? Has that impacted treatment at all? |
| Attitudes towards and perceptions of treatment | How do you feel about being on treatment? How are you finding treatment?  Do you have a process of taking treatment? Have you forgotten any pills?  Now that you are on treatment has anything changed compared to when you weren’t on it?  *Prompts:* Can you tell me a bit more about that? Is anything different physically? Mentally? How you interact with other people?  Is there anything you are worried or excited about with finishing treatment?  *Prompts:* What are you most looking forward to? Do you have any expectations of what finishing treatment will be like?  Do you feel supported being on treatment?  *Prompts:* Who supports you? In what ways? Is there any other support with hep C and treatment that you would want? Do you reckon there is anything that would make treatment easier for you? |
| Disclosure | Have you told anyone you are on treatment?  *Prompts:* Who do you tell? How do you feel about telling other people? What was that like? What did they reckon? |
| Relationship with healthcare and knowledge | How has the process been with the nurses or GP and treatment?  Is there anything you are still unsure about with treatment? |
| Behaviours and experiences | Being on treatment have you changed anything in your life or any behaviours?  *Prompts:* Has anything changed recently with your diet? Exercise? Lifestyle? Smoking? Alcohol? employment or how you feel about working? How you interact with people socially?  Can you tell me a little more about…?  What made you want to change… now?  Was it difficult to change…?  Do you reckon you will do anything differently after treatment?  Has the doctor told you anything you are meant to do while you are on treatment?  *Prompts:* Is there anything you have to do when you finish treatment?  Last time you talked about… how is that going? Has that changed at all? How do you feel about… |
| Information sharing | Have you told anyone else about treatment and how it’s going for you?  Has anyone asked you about treatment? What kinds of things are you telling other people?  How much do your peers and your circle talk about treatment?  *Prompts:* What do they say about it? What do you think about the stuff they’ve said? Why? If someone asked you about treatment what would you say? |
| Impact of hep C/ treatment | Do you notice you are on treatment? In what ways?  *Prompts:* Do you reckon treatment is working? How can you tell? |
| Reinfection | Are you worried about reinfection at all? Why/why not? |
| Conclusion | Next time we chat you’ll be finished treatment, any thoughts on what you are hoping that will be like?  Anything else you want to chat about? |

**Semi-structured flexible interview guide for the post-treatment interview**

| **Topic** | **Questions and prompts** |
| --- | --- |
| Introduction | So, to start with, how has your week been going?  *Prompts:* Do you want to just talk a bit about how thing are going for you at the moment?  Has anything changed for you since last time we spoke?  *Prompts:* Can you tell me a bit more about…? Has that impacted treatment at all? |
| Attitudes towards and perceptions of treatment | How do you feel about having finished treatment? How does it feel being cured?  What does it mean for you being cured? Emotionally? Socially? Physically?  Have you forgotten any pills?  Now that you have finished has anything changed compared to when you were still doing treatment?  *Prompts:* Can you tell me a bit more about that? Is anything different physically? Mentally? How you interact with other people?  Is there anything you are worried or excited now you have finished?  *Prompts:* What are you most looking forward to? |
| Disclosure | Have you told anyone you have finished treatment?  *Prompts:* Who do you tell? How do you feel about telling other people? What was that like? What did they reckon? |
| Relationship with healthcare | How has the process been with the nurses or GP and finishing treatment?  What is the process for you now? Is there anything you have to do? How do you feel about that? Is there anything you are unsure about? |
| Behaviours and experiences | Has finishing treatment changed anything in your life or any behaviours?  *Prompts:* Has anything changed recently with your diet? Exercise? Lifestyle? Smoking? Alcohol? employment or how you feel about working? How you interact with people socially?  Can you tell me a little more about…?  What made you want to change… now?  Was it difficult to change…?  Do you reckon you will do anything differently now that you don’t have hep C?  Last time you talked about… how is that going? Has that changed at all? How do you feel about… |
| Information sharing | Have you told anyone else about treatment and how it’s going for you?  Has anyone asked you about treatment? What kinds of things are you telling other people?  How much do your peers and your circle talk about hep C?  *Prompts:* What do they say about it? What do you think about the stuff they’ve said? Why? If someone asked you about treatment what would you say? |
| Impact of hep C | Do you notice that you have finished the treatment? In what ways?  *Prompts:* Do you reckon you can tell it’s gone? How? |
| Reinfection | Are you worried about reinfection at all? Why/why not? |
| Conclusion | Anything else you want to chat about? |
